# Leukocyte as an Independent Predictor of Lower-Extremity Deep Venous Thrombosis in Elderly Patients With Primary Intracerebral Hemorrhage

**DOI:** 10.3389/fneur.2022.899849

**Published:** 2022-07-12

**Authors:** Gang Wang, Wenjun Zhao, Zhiyong Zhao, Dengfeng Wang, Dong Wang, Ruobing Bai, Boru Hou, Haijun Ren

**Affiliations:** ^1^Department of Neurosurgery, Lanzhou University Second Hospital, Lanzhou, China; ^2^Key Laboratory of Neurology of Gansu Province, Lanzhou, China; ^3^Department of Health Management Center, Lanzhou University Second Hospital, Lanzhou, China

**Keywords:** deep vein thrombosis, inflammation, intracerebral hemorrhage, leukocytes, elderly

## Abstract

**Objective:**

Due to the interaction of leukocytes with platelets and coagulation factors, they may in turn play a role in hemostasis or the formation of thrombi. This study aimed to investigate the association of leukocytosis on admission with an increased risk of acute lower-extremity deep venous thrombosis (LEDVT) in elderly patients with primary intracerebral hemorrhage (ICH).

**Methods:**

This was a single-center, retrospective observational study of consecutive patients observed with spontaneous ICH aged 60 years or above at Lanzhou University Second Hospital from January 2017 to September 2021. Clinical data and demographic information were collected and analyzed. Univariate and multivariate analyses were conducted to identify independent risk factors of acute LEDVT. One-to-one matching was implemented to balance important patient characteristics by the groups' propensity score matching (PSM) analysis.

**Results:**

A total of 371 elderly patients with primary ICH fulfilled requirements for inclusion and exclusion, of whom 33 (8.89%) experienced LEDVT. Leukocyte counts were statistically higher in the LEDVT group compared to the non-LEDVT group [12.89 (8.80–14.61) × 10^9^ cells/L vs. 8.31 (6.60–10.75) × 10^9^ cells /L, *p* < 0.001]. Multivariate logistic regression models adjusted for several potential confounding factors were performed, and leukocytes were consistently a significant independent predictor of LEDVT. The optimal cut-off value of leukocyte counts calculated from the receiver operating characteristic (ROC) curve to predict LEDVT was 10.22 × 10^9^ cells /L (area under the curve:0.714, 95%CI 0.665–0.759; the sensitivity was 72.73%; the specificity was 71.01%) in elderly patients with primary ICH. After one-to-one PSM, compared to the matched non-LEDVT group, the matched LEDVT group had significantly higher leukocyte counts [11.98 (8.40–13.94) × 10^9^ cells/L vs. 6.12 (4.68–12.00) × 10^9^ cells/L, *p* = 0.003]. After PSM, the ROC curve was plotted for leukocytes as a predictor of LEDVT, with an AUC of 0.722 (95%CI 0.593–0.828, *p* = 0.001; the sensitivity was 87.10%, and the specificity was 61.29%). Elevated leukocytes remained independently significant as predictors of LEDVT in elderly patients with primary ICH.

**Conclusion:**

Leukocyte at admission is an independent risk factor of LEDVT in elderly patients with primary ICH.

## Introduction

Spontaneous intracerebral hemorrhage (ICH) is a devastating cerebrovascular disease with high morbidity, disability, and mortality (1, 2). Deep venous thrombosis (DVT) is a common but elusive illness that can lead to long-term disability or death, and DVT mainly occurs in the paralyzed lower extremities after ICH (3, 4). The incidence of post-stroke lower-extremity deep venous thrombosis (LEDVT) varies widely, from 10 to 75%, depending on the diagnosis method and the evaluation timing (5, 6). It is undeniable that LEDVT incidence is higher in acute spontaneous ICH than that in ischemic stroke (7). The literature has identified a series of risk factors that can predict LEDVT in ICH patients, including older age, paralysis, a history of deep vein puncture, immobilization, infection, and laboratory characteristics (5, 8, 9). Elderly patients with acute neurological illness, including acute ICH, are at high risk for LEDVT due to their increased prevalence of obesity, increased disease frequency, prolonged immobility, and increased levels of procoagulants without a corresponding increase in anticoagulants (10, 11). The detailed mechanism responsible for LEDVT in elderly patients with primary ICH is yet to be elaborated.

The leading laboratory test indicators to predict LEDVT are D-dimer, blood routine, and blood lipids (12–14). In recent years, the association of inflammatory biomarkers with LEDVT has become a research focus. Elevated inflammation indicators in critical illness, including spontaneous ICH, were considered independent risk factors for acute LEDVT patients, such as platelet to lymphocyte ratios and neutrophil-lymphocyte ratios (12, 14). Acute leukocytosis is a well-established phenomenon in ICH. Andrea et al. demonstrated that leukocytes were essential for regulating the coagulation cascade after acute ICH (15). Leukocytes interact with platelets and endothelial cells, and coagulation factors have been widely recognized as essential for promoting hemostasis under physiological and pathological conditions (16, 17). An acute leukocytosis may shift the hemostatic balance in favor of coagulation, thereby arresting bleeding after ICH (15). However, no study has examined the association between leukocytes and LEDVT in elderly patients with primary ICH.

Therefore, this study aimed to assess the incidence and risk factors associated with LE DVT in elderly patients with primary ICH and further analyze the correlation between leukocytes and LEDVT for early clinical prevention.

## Materials and Methods

### Study Population

This was a single-center, retrospective observational study of consecutive patients presenting with spontaneous ICH aged 60 years or above at Lanzhou University Second Hospital from January 2017 to September 2021. Our local Ethics Committee approved the study. Patients were eligible, if (1) diagnoses of ICH required confirmation by computed tomography (CT) scan within 48 h after admission; (2) peripheral blood samples were obtained through venipuncture upon admission; (3) the diagnosis of LEDVT was confirmed by Doppler ultrasonography. Exclusion criteria were listed as follows: (1) age < 60 years; (2) diagnoses with secondary ICH, including aneurysm, arteriovenous malformation, Moyamoya disease, brain tumor, and hemorrhagic infarction; (2) primary intraventricular hemorrhage (IVH); (3) historical stroke; and (4) history of DVT or pulmonary embolism (PE), history of thrombophilia.

### Baseline Data Collection

Patients' demographic information, medical history, diagnostic information, laboratory data, length of intensive care unit (ICU) stay, intervening measures (conservative therapy and surgery), and comorbidities at the hospital (pulmonary infection) were collected *via* an electronic medical record system. Peripheral venous blood was obtained by venous puncture within 1 h of admission for laboratory examinations, including blood routine, blood biochemical indexes, D-dimer, and blood coagulation. Leukocyte counts (reference range, 3.5 × 10^9^ cells/L to 9.5 × 10^9^ cells/L) and hemoglobin (reference range, 115–150 g/L) were collected from the blood routine test. ICH hematoma volume was measured on the initial CT using the ABC/2 method. ICH location on admission CT scan was grouped into 2 categories, namely, patients with lobar ICH and those with deep ICH. Hematoma locations at the cortical and subcortical junction were defined as lobar ICH, while those involving the thalamus, basal ganglia, brainstem, and cerebellum were described as deep ICH (18, 19).

Pulmonary infection was defined as an episode of infection that occurred more than 48 h after hospital admission (20). According to the previous literature, the pulmonary infection can be diagnosed clinically, microbiologically, and radiologically (21, 22).

### Diagnosis of LEDVT

Color Doppler ultrasonography was routinely performed once weekly for patients admitted to the neurological ICU, every 2 weeks for patients admitted to the neurosurgery unit, and as indicated for patients with signs and symptoms of LEDVT (e.g., swelling and pain) (14, 23). In patients with signs or symptoms, LEDVT was diagnosed by complete ultrasound with Doppler waveforms and images from thigh to ankle, and based on lower extremity ultrasonography showing incompressible distal or proximal veins (24). When the deep vein was completely embolized, no blood flow was detected at the lesion, and the distal blood flow did not increase when squeezed. The probe detected the blood flow signal filling the defect when the deep vein was partially embolized. A small blood flow was visible only after squeezing the distal limb (14).

The Caprini risk assessment score was utilized to strengthen the observance of venous thromboembolism prophylactic regimens for patients under both medical and surgical conditions and accordingly identify the required thromboprophylaxis mode. Prevention of venous thrombosis, including LEDVT, such as getting out of bed, is based on the Caproni score (25). A lower-extremity pneumatic pump was used to prevent LEDVT. Low-dose subcutaneous heparin (100 U/kg, QD) was used to prevent deep vein thrombosis (26, 27). Low-molecular-weight heparin (100 U/kg, Q12H) was applied in primary ICH patients with acute LEDVT as soon as the venous Doppler ultrasound confirmed the diagnosis.

### Statistical Analysis

All statistical analyses were conducted with the SPSS software (version 25.0, IBM SPSS, IBM Corp, USA), Prism 8.3.0 (GraphPad Software, San Diego, CA, USA), and MedCalc version 20.0.4 (MedCalc Software, Ostend, Belgium). The χ^2^ test, Fisher's exact test, and the Mann-Whitney U test were compared. The box plots graph represents the median with an interquartile range (IQR). For correlation analysis, Spearman's correlation was applied. All variables with *p* < 0.10 in univariable analysis were selected for the multivariate logistic regression to evaluate independent risk factors. The multivariate logistic regression models were used to determine the relationship of leukocytes with LEDVT. Model 1 was unadjusted. Model 2 was adjusted by baseline GCS, leukocyte counts, baseline volume, length of ICU stay, treatment, pulmonary infection, and prophylactic use of low-dose subcutaneous heparin. Model 3 = Model 2 and adjusted by neutrophils. We constructed the receiver operating characteristic (ROC) curve and calculated the area under the ROC curve (AUC) to access the predictive power of leukocytes for LEDVT. To match baseline characteristics, propensity score matching (PSM) analysis was performed to obtain a 1:1 nearest-neighbor matching, with a match tolerance of 0.01. Variables with statistical significance (*p* < 0.05) in the univariate analysis were included in the matching. A two-sided *p*< *0.05* was determined to be statistically significant.

## Results

### Baseline Characteristics of the Study Patients

**Figure 1:**
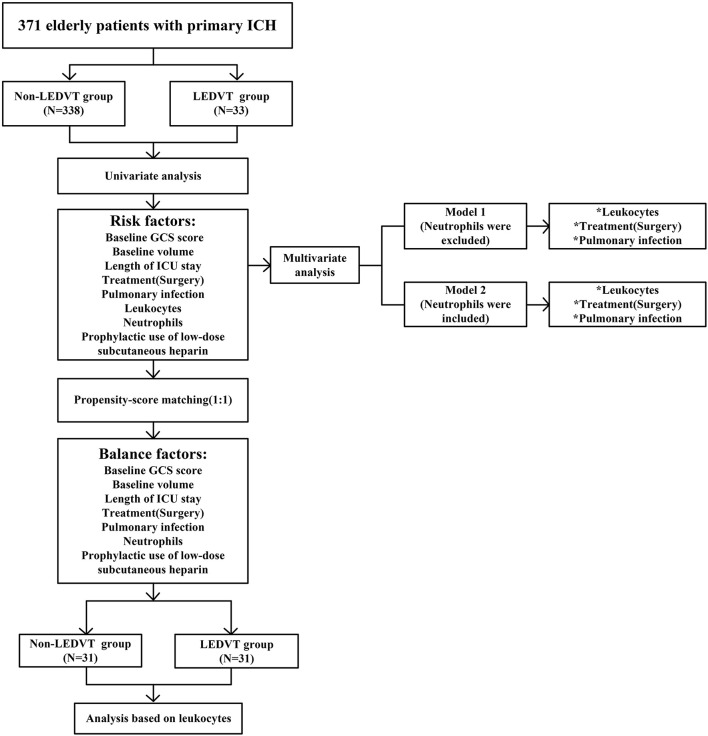
Flowchart of the study. LEDVT, lower-extremity deep venous thrombosis; GCS, Glasgow Coma Scale; ICH, intracerebral hemorrhage; ICU, intensive care unit. *p < 0.05.

**Figure 2:**
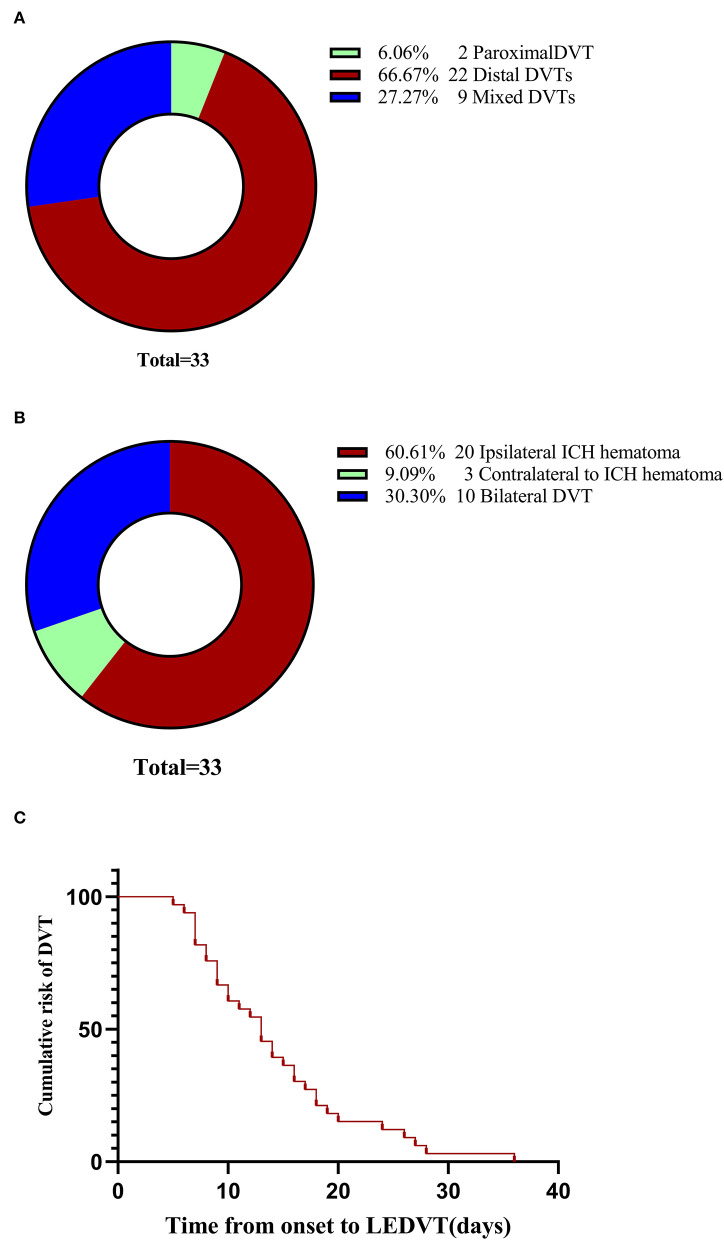
LEDVT overall. **(A)** Pie chart showing the number and percentage of the three types of LEDVT. **(B)** Pie chart demonstrating the distribution, number, and percentage of LEDVT relative to the location of intracerebral hemorrhage hematoma. **(C)** Cumulative LEDVT rate using Kaplan-Meier analysis from intracerebral hemorrhage onset to LEDVT. LEDVT, lower-extremity deep venous thrombosis.

The study flow is documented in Figure 1. A total of 371 elderly patients with primary ICH fulfilled requirements for both inclusion and exclusion, of whom 33 (8.89%) experienced LEDVT, 22 distal DVTs (66.67%), 9 mixed DVTs (27.27%), and 2 proximal DVTs (6.06%) (Figure 2A). Figure 2B illustrates the distribution, number, and percentage of LEDVT relative to the location of ICH hematoma. In the LEDVT group, pulmonary embolism occurred in four cases. The cumulative LEDVT rate using Kaplan-Meier analysis from ICH onset to LEDVT is displayed in Figure 2C. The time from ICH onset to LEDVT was 13.0 (IQR,8.50–18.0) days. The median leukocyte counts were [8.57 (IQR 6.77–11.37), × 10^9^ cells/L]. The baseline GCS score was 11 (IQR, 8.0–13.0), and the initial ICH volume was 14.15 ml (IQR, 4.93–35.76 ml). A total of 224 patients (60.4%) were treated conservatively, and 147 patients (39.6%) had surgery.

### Correlation Between Peripheral Blood Leukocyte Counts and Initial Clinical Status at Admission

**Figure 3:**
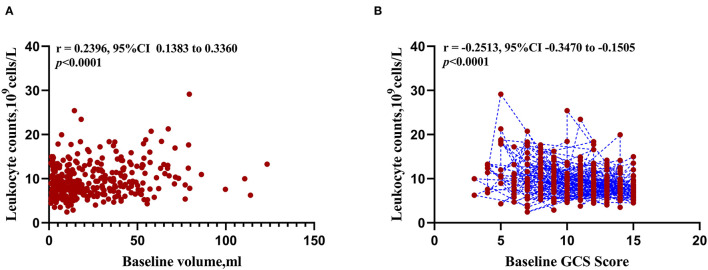
Correlation between peripheral blood leukocyte counts and initial clinical status at admission. **(A)** Scatterplot of expression correlation between leukocyte counts and baseline volume. **(B)** Scatterplot of expression correlation between leukocyte counts and baseline Glasgow Coma Scale score.

The results of correlation analysis demonstrated that leukocyte was positively correlated with the initial ICH volume (*r* = 0.2396, 95%CI 0.1383 to 0.3360, *p* < 0.001, Figure 3A) and negatively correlated with the baseline GCS score (*r* = −0.2513, 95%CI−0.3470 to−0.1505, *p* < 0.001, Figure 3B).

### Association of Peripheral Blood Leukocyte Counts With LEDVT

**Figure 4:**
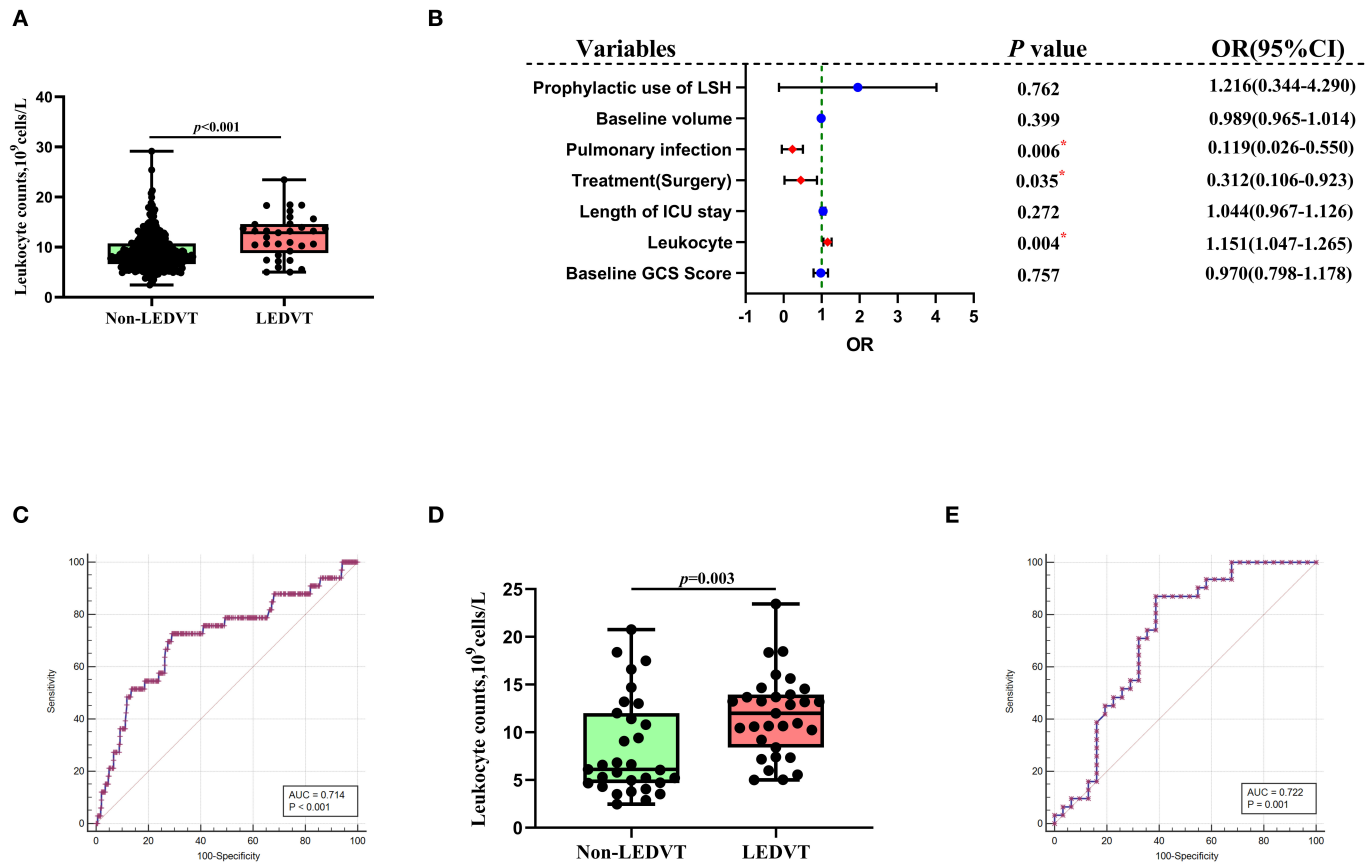
Association of peripheral blood leukocyte counts with LEDVT. **(A)** Comparison of leukocyte counts between Non-LEDVT and LEDVT groups. **(B)** Forest plot of multivariate analysis for risk factors associated with LEDVT. **(C)** Receiver operating curve analysis of leukocyte counts for predicting LEDVT. The optimal cutoff value for leukocyte counts as a predictor for LEDVT in primary intracerebral hemorrhage patients was determined to be 10.22 × 10^9^ U/L (AUC was 0.714, the sensitivity was 72.73%, and the specificity was 71.01%). **(D)** Comparison of leukocyte counts between non-LEDVT and LEDVT groups after propensity-score matching. **(E)** The area under the curve of leukocytes for LEDVT was 0.722 with a sensitivity of 87.10% and a specificity of 61.29%. A median with the interquartile range was shown for box plots graph in panel A and panel D. Groups were compared using Mann-Whitney U tests. Correlations were determined using Spearman's correlation analysis (*p< *0.05*). LEDVT, lower-extremity deep venous thrombosis; LSH, low-dose subcutaneous heparin; OR, Odds ratios; CI, Confidence interval.

Univariate and multivariate analyses were conducted to identify independent predictors of LEDVT in elderly patients with primary ICH. Demographic and clinical features between Non-LEDVT and LEDVT groups were compared, as depicted in Table 1. Baseline GCS score (*p* < 0.001), baseline ICH volume (*p* = 0.005), length of ICU stays (*p* = 0.007), surgery (*p* < 0.001), pulmonary infection (*p* < 0.001), neutrophils (*p* = 0.004), prophylactic use of low-dose subcutaneous heparin (*p* < 0.001), and leukocyte (*p* < 0.001) were found to have a statistically significant association with the occurrence of LEDVT in elderly patients with primary ICH. Leukocyte counts were statistically higher in the LEDVT group compared to the non-LEDVT group [12.89 (8.80–14.61) × 10^9^ cells/L vs. 8.31 (6.60–10.75) × 10^9^ cells/L, *p* < 0.001; Table 1; Figure 4A]. Leukocytes were positively associated with the occurrence of LEDVT with an OR value of 1.184 [95% confidence interval (CI) 1.091–1.284, p < 0.001) in the unadjusted model (Model 1, Table 2). The odds ratios (ORs) were adjusted for all outcomes on the baseline GCS, leukocyte counts, baseline volume, length of ICU stay, treatment, pulmonary infection, prophylactic use of low-dose subcutaneous heparin, and neutrophils. After adjustment in the multivariate model (Model 2, Table 2), leukocyte [OR 1.151, 95%CI 1.047–1.265, *p* = 0.004], treatment (surgery) (OR 0.312, 95%CI 0.106–0.923, *p* = 0.035), and pulmonary infection (OR 0.119, 95% CI 0.026–0.550, *p* = 0.006) were statistically associated with LEDVT (Table 2; Figure 4B). The forest plot (Figure 4B) displays that the leukocyte was an independent risk factor for LEDVT in elderly patients with primary ICH. The optimal cut-off value of leukocyte counts calculated by the ROC curve to predict LEDVT was 10.22 × 10^9^/L (AUC:0.714, 95%CI 0.665–0.759, *p* < 0.001; the sensitivity was 72.73%, and the specificity was 71.01%; Figure 4C) in elderly patients with primary ICH. To explore the relationship between neutrophils and LEDVT, neutrophils were included in a multivariate analysis model (Model 3 = Model 2 + neutrophils, Table 3). Interestingly, leukocytes remained associated with the occurrence of LEDVT. Overall, leukocytes were positively associated with the occurrence of LEDVT with an OR of 1.184 (95%CI 1.091-1.284), 1.151 (95%CI 1.047–1.265), and 1.483 (95%CI 1.092–2.014) in Model 1, Model 2, and Model 3, respectively.

**Table 1:** Univariate analysis of association with LEDVT before and after propensity-score matching in spontaneous intracerebral hemorrhage patients.

**Characteristics**	**Before propensity-score matching**			**After propensity-score matching**		
	**Non-LEDVT**	**LEDVT**	***P* value**	**Non-LEDVT**	**LEDVT**	***P* value**
	**(*N* = 338)**	**(*N* = 33)**		**(*N* = 31)**	**(*N* =31)**	
Age, yrs, median (IQR)	67.0 (64.0–72.0)	68.0 (63.0–72.0)	0.557	68.0 (63.0–75.0)	67.0 (63.0–71.0)	0.425
**Gender** (*N*, %)			0.951			
Male	203 (60.1)	20 (60.6)		21 (67.7)	20 (64.5)	0.788
Female	135 (39.9)	13 (39.4)		10 (32.3)	11 (35.5)	
**Medical history**						
Hypertension (*N*, %)	228 (67.5)	24 (72.7)	0.536	26 (83.9)	22 (71.0)	0.224
Diabetes (*N*, %)	61 (18.0)	5 (15.2)	0.678	8 (25.8)	5 (16.1)	0.349
Smoking (*N*, %)	33 (9.8)	5 (15.2)	0.330	5 (16.1)	5 (16.1)	1.000
Alcohol (*N*, %)	36 (10.7)	5 (15.2)	0.431	6 (19.4)	4 (12.9)	0.490
Prior anticoagulation or antiplatelet therapy (*N*, %)	26 (7.7)	4 (12.1)	0.373	3 (9.7)	3 (9.7)	1.000
**Admission vital signs**						
Systolic blood pressure, median (IQR)	156 (147–170)	156 (150–162.5)	0.987	159.0 (151.0–170.0)	156.0 (150–162.0)	0.278
Diastolic blood pressure, median (IQR)	89 (78–98)	86 (79.50–98)	0.864	89.0 (82.0–102.0)	86.0 (80.0–96.0)	0.714
Time from symptom onset to initial CT, hours, median (IQR)	4.0 (3.0–5.0)	4.0 (3.0–5.0)	0.228	5.0 (3.0–5.0)	4.0 (3.0–5.0)	0.756
Baseline Glasgow coma score, median (IQR)	11.0 (9.0–14.0)	9.0 (7.0–11.0)	<0.001	8.0 (7.0–11.0)	9.0 (7.0–11.0)	0.570
Baseline volume, ml, median (IQR)	12.64 (4.32–34.10)64(	(27.36 (14.43–47.82)	0.00.00505	29.34 (12.50–38.54)	25.86 (13.78–49.17)	0.741
Presence of intraventricular hemorrhage (*N*, %)	83 (24.6)	11 (33.3)	0.269	10 (32.3)	10 (32.3)	1.000
ICH location (*N*, %)			0.141			0.279
Lobar	63 (18.6)	3 (9.1)		6 (19.4)	3 (9.7)	
Deep	275 (81.4)	30 (90.9)		25 (80.6)	28 (90.3)	
**Admission laboratory**						
Leukocyte counts ,10^9^cells/L, median (IQR)	8.31 (6.60–10.75)	12.89 (8.80–14.61)	<0.001	6.12 (4.68–12.00)	11.98 (8.40–13.94)	0.003
Neutrophils counts,10^9^cells/L, median (IQR)	6.86 (4.79–9.37)	10.17 (5.80–12.77)	0.004	4.49 (3.47–10.67)	10.12 (5.59–12.05)	0.042
Hemoglobin, g/L, median (IQR)	148.50 (136.0–162.0)	150.0 (137.0–159.0)	0.979	148.0 (129.0–162.0)	150.0 (136.0–161.0)	0.725
Platelet,10^9^/L, median (IQR)	162.50 (124.0–200.25)	155.0 (105.50–196.50)	0.391	129.0 (100.0–170.0)	155.0 (103.0–196.0)	0.248
Prothrombin time, seconds, median (IQR)	11.40 (11.00–12.13)	11.40 (10.90–11.95)	0.383	11.50 (11.20–11.90)	11.30 (10.80–12.00)	0.212
International normalized ratio, median (IQR)	1.02 (0.95–1.08)	1.01 (0.96–1.08)	0.754	1.02 (0.98–1.06)	1.00 (0.96–1.08)	0.464
Activated partial thromboplastin time, seconds, median (IQR)	25.30 (21.50–28.83)	25.60 (21.35–30.40)	0.742	25.20 (22.20–30.40)	24.40 (21.20–30.30)	0.714
Fibrinogen, g/L, median (IQR)	3.0 (2.50–3.51)	2.89 (2.47–3.60)	0.805	3.04 (2.70–3.70)	2.90 (2.44–3.60)	0.382
D–dimer, ug/ml, median (IQR)	0.57 (0.3–1.13)	0.78 (0.37–1.80)	0.119	0.60 (0.27–0.99)	0.72 (0.36–1.54)	0.210
Length of ICU stay, days, median (IQR)	0.0 (0.0–4.0)	5.0 (0.0–14.0)	0.007	8.0 (0.0–15.0)	0.0 (0.0–14.0)	0.228
Prophylactic use of low-dose subcutaneous heparin, (*N*, %)	30 (8.9)	7 (21.2)	<0.001	9 (29.0)	6 (19.4)	0.374
Treatment			<0.001			0.520
Conservative therapy (*N*, %)	217 (64.2)	7 (21.2)		5 (16.1)	7 (22.6)	
Surgery (*N*, %)	121 (35.8)	26 (78.8)		26 (83.9)	24 (77.4)	
Pulmonary infection (*N*, %)	177 (52.4)	31 (93.9)	<0.001	29 (93.5)	29 (93.5)	1.000

*CT, computed tomography; LEDVT, lower-extremity deep venous thrombosis; GCS, Glasgow Coma Scale; ICH, intracerebral hemorrhage; IQR, Interquartile range; ICU, intensive care unit*.

**Table 2:** Predictors for LEDVT of spontaneous intracerebral hemorrhage in the multivariate model.

	**(Model 1)**^**a**^ **Unadjusted OR (95%CI)**				**(Model 2)**^**a**^ **AdjustedAOR (95%CI)**			
**Independent variable**	**OR**	**Lower**	**Upper**	***P*-value**	**OR**	**Lower**	**Upper**	***P*-value**
Baseline GCS	0.821	0.729	0.923	0.001	0.970	0.798	1.178	0.757
Leukocyte	1.184	1.091	1.284	<0.001	1.151	1.047	1.265	0.004*
Baseline volume	1.017	1.003	1.031	0.015	0.989	0.965	1.014	0.399
Length of ICU stay	1.112	1.058	1.168	<0.001	1.044	0.976	1.126	0.272
Treatment (surgery)	0.150	0.063	0.356	<0.001	0.312	0.106	0.923	0.035*
Pulmonary infection	0.071	0.017	0.301	<0.001	0.119	0.026	0.550	0.006*
Prophylactic use of low-dose subcutaneous heparin	0.362	0.145	0.903	0.029	1.216	0.344	4.290	0.762

^a^Adjusted covariates: Model 1: unadjusted; Model 2: baseline GCS, leukocyte counts, baseline volume, length of ICU stay, treatment, pulmonary infection, and prophylactic use of low-dose subcutaneous heparin. CI, Confidence interval; LEDVT, lower-extremity deep venous thrombosis; GCS, Glasgow Coma Scale; ICU, intensive care unit; OR, Odds ratios. *p < 0.05.

**Table 3:** Predictors for LEDVT of spontaneous intracerebral hemorrhage in the multivariate model.

		**(Model 3)** ^**a**^**Adjusted** **AOR (95%CI)**		
**Independent variable**	**OR**	**Lower**	**Upper**	***P*-value**
Baseline GCS	0.991	0.813	1.207	0.926
Leukocyte	1.483	1.092	2.014	0.012*
Neutrophils	0.770	0.572	1.036	0.084
Baseline volume	0.991	0.966	1.016	0.469
Length of ICU stay	1.044	0.967	1.127	0.273
Treatment (surgery)	0.322	0.107	0.966	0.043*
Pulmonary infection	0.113	0.024	0.531	0.006*
Prophylactic use of low-dose subcutaneous heparin	1.241	0.346	4.454	0.740

^a^Adjusted covariates: Model 3 : Model 2 + neutrophils. CI, Confidence interval; LEDVT, lower-extremity deep venous thrombosis; GCS, Glasgow Coma Scale; ICU, intensive care unit; OR, Odds ratios. *p < 0.05.

In order to reduce the effects of confounding factors, we conducted a PSM. One-to-one PSM yielded 62 patients, with 31 patients in each group. No statistical significance was detected in the baseline GCS score, baseline ICH volume, length of ICU stays, received treatment, and pulmonary infection between the two groups. Compared to the matched non-LEDVT group, the matched LEDVT group had significantly higher leukocyte counts [11.98 (8.40–13.94) × 10^9^ cells/L vs. 6.12 (4.68–12.00) × 10^9^ cells/L, *p* = 0.003; Table 1; Figure 4D]. After PSM, the ROC curve was plotted for leukocytes as a predictor of LEDVT, with an AUC value of 0.722 (95%CI 0.593–0.828, *p* = 0.001; the sensitivity was 87.10%, and the specificity was 61.29%; Figure 4E). Elevated leukocytes remained independently significant as predictors of LEDVT in elderly patients with primary ICH. We had an interesting finding that a statistically significant correlation was still observed between neutrophils and LEDVT (*p* = 0.042, Table 1), although neutrophils were included in the PSM.

## Discussion

In this study, we applied multivariate and PSM analyses to explore the association of admission leukocyte counts with LEDVT in elderly patients with primary ICH. The significant findings of this study may be summarized as follows: (1) the elevated leukocyte level was a significant independent risk factor of LEDVT; (2) leukocyte counts were inversely associated with the baseline GCS score; (3) leukocyte counts were positively correlated with the baseline ICH volume. The optimal cut-off value of leukocyte counts calculated from the ROC curve to predict LEDVT was 10.22 × 10^9^/L in elderly patients with primary ICH. After reducing the effects of confounding factors with a PSM analysis, the matched LEDVT group had significantly higher leukocyte counts than the matched non-LEDVT group. To the best of our knowledge, this is the first study investigating the relationship between admission leukocytes and LEDVT in elderly patients with primary ICH.

Shortly after the onset of acute ICH, the hematoma component triggers an inflammatory response that together with the hematoma, aggravates brain tissue damage and further increases inflammatory cells in peripheral blood (28, 29). When the blood-brain barrier is compromised after ICH, leukocytes and inflammatory agents can infiltrate into perihematomal areas and eventually cause leukocyte elevation through cerebrospinal fluid circulation (30). The Spearman correlation analysis revealed a weak positive correlation between leukocytes and the initial ICH volume (*r* = 0.2396). The correlation analysis can only provide associations and cannot draw inferences regarding a causal relationship between leukocytes and the baseline ICH volume. The previous literature suggested that the higher admission leukocyte count was negatively associated with hematoma expansion (15), supporting a possible link between acute inflammatory, leukocyte, and coagulation. Results of this study support this inference and extend it, suggesting that inflammation and leukocyte activation are implicated in coagulation after ICH (15, 31, 32).

Thrombosis is a complex process involving factors related to coagulation and inflammation. Inflammation significantly contributes to LEDVT (12–14, 33, 34). Inflammation may affect various stages of the coagulation pathway. Subsequently to acute inflammation, the coagulation cascade is triggered, leading to thrombotic events. Aggregation of leukocytes exacerbates thrombosis (35). The LEDVT group showed increased leukocyte counts compared with the non-LEDVT group, in this study. Leukocytes interact with platelets, endothelium, and coagulation factors and thus may play a vital role in the pathophysiology of LEDVT by regulating the coagulation system (35, 36).

A systemic inflammatory response occurs in acute ICH, and peripheral blood leukocytes, particularly neutrophils, are elevated. Activated neutrophils exhibit significant procoagulant properties. Animal models of DVT demonstrate that neutrophils stimulate thrombosis and promote the coagulation process by forming neutrophil extracellular traps, resulting in abundant deep vein thrombosis (37, 38). In this study, multivariate analysis showed no significant association between neutrophils and LEDVT. Interestingly, although neutrophils were included in the PSM, a statistically significant correlation was still observed between neutrophils and LEDVT after PSM. The inconsistency of our results might be related to the relatively small sample size. More high-quality studies with large sample sizes are needed to clarify this issue.

A complex combination of interactions among the various components in the leukocytes may result in the occurrence of LEDVT. Approximately 50–70% of white blood cells are neutrophils, which comprise human blood's most abundant type of leukocyte. In the view of the mechanism, Yago et al. (34) confirmed that neutrophils cooperatively signal through glycoprotein ligand-1 and CXCR_2_ to promote DVT. First, neutrophils alter the coagulation balance by significantly expressing and releasing tissue factors, thereby favoring thrombosis (39). Second, neutrophils indirectly increase active tissue factors, downregulate tissue factor pathway inhibitors, and promote thrombus formation (40). Third, neutrophil extracellular traps can activate platelets, factor X, and factor XII, enhance thrombin generation, and help stabilize fibrin clots (38, 41). Activating neutrophils during acute ICH may place the body in a procoagulant state (15). Fourth, neutrophils in white blood cells are sticky and allow a developing thrombus to adhere to the walls of blood vessels (38). Sticky neutrophils trigger further endothelial damage and activation of the coagulation cascade, exacerbating thrombus formation (42). Fifth, although thrombus formation requires neutrophils, monocytes further augment thrombosis by expressing tissue factors (34). Intimate associations exist between inflammation and thrombosis, with inflammatory states promoting coagulation and thrombus amplifying inflammation (43). Remarkably, systemic inflammation amplifies the coagulation process, and coagulation can worsen inflammatory progression, especially in elderly patients with ICH, predisposing them to the progression of LEDVT events (3).

Virchow's triad, first described in 1856, summarizes the numerous risk factors for DVT into three essential elements contributing to thrombosis, venous stasis, vascular injury, and a hypercoagulable state (10, 35). The clinical conditions following primary ICH most closely associated with LEDVT are fundamentally related to elements of Virchow's triad, including acute critical illness stress, prolonged immobility, advancing age, and invasive endovascular procedures (7, 44–46). First, aging is associated with changes in the levels of coagulation factors and fibrinolytic proteins, which activate the coagulation cascade. Older age was the most significant risk factor for developing LEDVT (5). Second, elderly patients are bedridden for a long time after ICH, especially limb paralysis caused by ICH, which further aggravates blood stasis and eventually forms LEDVT (47). The previous literature reported a high risk of LEDVT in hospitalized elderly patients with intracerebral hemorrhage, up to 66.7% (7). The longer they stay still, the higher the risk of developing a LEDVT. Third, during surgery or when intravenous catheters are inserted in patients with ICH, it may damage the vascular endothelium or expose subendothelial factors and reduce blood flow in the blood vessels (48), thereby activating a series of the coagulation cascade, and ultimately a thrombotic event occurs.

Our study revealed significant correlations between the leukocyte levels, baseline GCS score, and initial ICH volume, indicating that the leukocyte levels could reflect ICH severity. Consistent with prior reports, leukocytes are associated with ICH severity (6, 15). Although the GCS score and initial ICH volume were significantly related to leukocyte levels, the GCS score and initial ICH volume were not risks for LEDVT, suggesting that the GCS score and initial ICH volume were only indicators of the severity of the disease. Bembenek et al. (49) also demonstrated that the National Institute of Health Stroke scale, reflecting acute stroke severity, was not a risk factor for LEDVT.

### Limitations

Our single-center retrospective study has several limitations. First, our study has inherent limitations of this type of design (i.e., the impossibility of establishing definitive causal connections) and might limit the generalizability of the findings. Second, the sample size was relatively small due to the rigorous selection standards. Future studies with a larger sample size would allow for more rigorous validation. Third, only baseline values of leukocyte counts were analyzed rather than the temporal trend. Clinically, LEDVT formation and diagnosis may take a long time. Therefore, attention should be paid to the relationship between the dynamic changes of leukocytes and the occurrence and development of LEDVT. Fourth, it is also worth noting that the actual incidence of LEDVT may be higher than that reported in this study. In neurosurgery wards, lower extremity vascular ultrasonography was not routinely performed weekly, and Doppler ultrasonography was usually performed when the patient was symptomatic. This might have led to an underestimate of the prevalence of LEDVT. Finally, phlebography may diagnose more LEDVT and earlier smaller clots. Due to the severity of the ICH, we did not routinely include venography.

## Conclusion

Elevated leukocyte upon admission is an independent risk factor of acute LEDVT in elderly patients with primary ICH. Leukocytes are cheap, rapid, convenient, and widely available biomarkers that may improve our ability to identify and stratify early LEDVT risk in clinical practice. Further multicenter randomized studies are needed to investigate the association between leukocyte dynamics and LEDVT.

## Data Availability Statement

The raw data supporting the conclusions of this article will be made available by the authors, without undue reservation.

## Ethics Statement

The studies involving human participants were reviewed and approved by the Ethics Committee of Lanzhou University Second Hospital. The ethics committee waived the requirement of written informed consent for participation.

## Author Contributions

GW and WZ designed the study and drafted the manuscript. GW, ZZ, and DeW collected and analyzed data. DoW and RB helped in the statistical analysis and prepared the figures. BH supervised the study and revised the manuscript. GW, BH, and HR initiated and organized this study. All authors reviewed and edited the manuscript and approved the final manuscript.

## Funding

This study was supported by the Natural Science Foundation of Gansu Province (Grant Numbers: 20JR5RA336 and GSWSKY2021-006), and the Cuiying Scientific and Technological Innovation Program of Lanzhou University Second Hospital (Grant Number: CY2021-MS-B10).

## Conflict of Interest

The authors declare that the research was conducted in the absence of any commercial or financial relationships that could be construed as a potential conflict of interest.

## Publisher's Note

All claims expressed in this article are solely those of the authors and do not necessarily represent those of their affiliated organizations, or those of the publisher, the editors and the reviewers. Any product that may be evaluated in this article, or claim that may be made by its manufacturer, is not guaranteed or endorsed by the publisher.
